# Effects of overlayer capping and lattice strain on perpendicular magnetic anisotropy of TM|FePt|MgO heterostructures

**DOI:** 10.1038/s41598-018-27424-y

**Published:** 2018-06-21

**Authors:** Xiaocui Han, Hong Cui, Bo Liu, Cunling Tian, Junzhong Wang, Hong Chen, Hongkuan Yuan

**Affiliations:** 1 School of Physical Science and Technology, Southwest University, Chongqing, 400715 People’s Republic of China; 2 School of Mechanical Engineering, Shannxi University of Technology, Shannxi, 723001 People’s Republic of China

## Abstract

Magnetic tunnel junctions (MTJs) with ferromagnetic electrodes possessing the strong perpendicular magnetocrystalline anisotropy (PMA) are of great interest as they have a potential for realizing next-generation high-density non-volatile memory and logic chips. To date, it is an urgent and critical issue to continuously promote the PMAs through feasible modifications such as the substitution of ferromagnetic layers as well as the overlayer coating on them. Here, we perform the relativistic first principles calculations of TM|*L*1_0_-FePt|MgO sandwich systems, and demonstrate that the changes in PMAs by capping TM layers are always giant and positive, e.g., PMA of Fe|FePt|MgO, the largest one among all our studied systems, is about 2 times larger than that of FePt|MgO. The interfacial PMAs at TM|FePt and FePt|MgO interfaces are extracted to be 3.31~9.40 meV and 3.32 meV, respectively, which are at least 3 times larger than 0.93 meV/ML of interior FePt layer. We illustratively verify that PMAs of TM|FePt|MgO can be turned in a large range by varying the TM layer and in-plane strain. Our results and model analyses provide useful insights for how these magnetic quantities are linked, and pave a way for the promotion of PMAs of FePt-based heterostructures via contact with TM overlayers.

## Introduction

Perpendicular magnetic anisotropy (PMA) is of great importance in building high density magnetic data storage devices such as spin-transfer-torque magnetic random access memory (STT-MRAM)^1,2^, because the strong PMA can not only improve the data storage area density and the thermal stability but also reduce the current density for magnetization reversal. Since the breakthrough discovery of the large interfacial PMA in CoFeB|MgO (Ta|CoFeB|MgO) heterostructures^3^ which was designated as the ferromagnetic electrode models of magnetic tunnel junction (MTJ), intensive investigations have been done on various heterostructures of transition-metal|ferromagnic-metal|insulating MgO (TM|FM|MgO)^2^.

So far, it has been well recognized that the significant PMA in MgO-heterostructures is originated from the strong interfacial hybridization between Fe(Co)-3*d* and O-2*p* orbitals at the contacted FM and MgO interface^2–7^. The subsequent researches have pointed out that an appreciative TM overlayer could further strengthen the PMA and improve other desired magnetic properties through the control of the TM|FM interface. For example, recent experiment has approved that a capping Ta layer coating on CoFeB|MgO is essential to enhance the PMA and the tunnel magnetoresistance ratio as well as to reduce the switching current of current-induced magnetization^3^. Along with these works, the PMA was found to increase from 1.8 erg/cm^2^ to 1.9 (2.3) erg/cm^2^ as the Ta layer was replaced with Ir (Hf) layer^5,8–12^. Other attempts have been made on analogous systems such as FeCo|MgO^13–15^, Fe(Co)|MgO^16–21^, TM|Fe^18,22,23^, X|CoFeB|MgO (X = Mo^24,25^, Ru^25–27^, Ta^25–28^, W^25,26,29^, Hf^25,27,30^, Pt^25,30^, and other TM^25,26,30^). Although the interfacial PMA in the heterostructures within MgO layers are experimentally very high^31^, it is still insufficient for practical applications. Typically, the PMA in Ta|CoFeB|MgO films rapidly decreases with the annealing temperature higher than 300 °C, which will degrade their performance in the MTJ^32,33^.

Toward the successful perpendicular MTJ devices, one favorable avenue to maximize the PMA is the modelling of prototype ferromagnets beyond soft-magnetic CoFe alloys. Hard-magnetic material with intrinsically high PMA is a promising candidate. In this content, 3*d*–5*d* alloys or their layers such as Fe(Co)-Pt with face-centered tetragonal (fct) *L*1_0_ phase^34–36^ were found to have the large magnetic anisotropy (*K*_u_~7 × 10^7^ ergs/cm^3^) and the uniaxial easy axis (along the direction of crystallographic *c* axis)^37^. Experimentally, previous measurements of the FePt|MgO heterostructures have shown that MgO is propitious to promote the formation of (001) texture in FePt thinfilms due to their small lattice mismatch within 8.5%. This can easily preserve the perpendicular anisotropy^38^ and would exhibit the large tunnel magnetoresistance (TMR)^39^. In addition, the PMA and coercivity modulated by electric field have been also reported^40–42^, where the PMA increases in Pt|FePt^40^ but decreases in Au|FePt|MgO^42^ as electrons are depleted from FePt layer. Theoretically, Taniguchi *et al*.^43^ have found that the Fe-terminated interfaces will result in higher TMR ratios (380%) than the Pt-terminated interfaces (70%). Cuadrado and Chantrell^44^ have proposed an enhancement of the interface magnetic moments and a stronger chemical bonding, which is attributed to significant overlap between the Fe-3*d* bands and the O-2*p* orbitals. Combined DFT calculations and micromagnetic simulations, Zhu *et al*.^45–47^ have successively explored the Pt(Cu)|FePt|MgO-based perpendicular MTJ, focusing on the effects of epitaxial strain, ferromagnetic thickness, interfacial interaction, electric field, and switching-current on the PMA as well as the magnetization switching. Ultimately, they found that the strain/thickness effect has significant/less influence on the PMA, and the PMA varies linearly with the change of the electric field (the critical switching current *J*_*c*_ linearly increases with the decreasing in-plane lattice parameter *a*).

Despite these investigations, underlying mechanism regarding on the PMA is still remained elusively at the electronic structure level. Since the interactions at TM|FePt interface may result in large interfacial PMA as it does in the TM|CoFeB heterostructure^3–10^, it is essential to quantitatively extract how much the TM|FePt interface would present and to make a comparison with the PMAs of FePt layers and the Fe|MgO interface. Therefore, we systematically report the magnetic results of the TM|FePt|MgO heterostructures by using a wide range of transition metal elements (3*d*-TM: Fe, Ni, Cu; 4*d*-TM: Rh, Pd, Ag; 5*d*-TM: Ir, Pt, Au) as the overlayers that are individually contacted with the FePt slab. Since the FePt thickness has been proved to play a relative weak impact on magnetic moments and PMAs of the FePt thinfilm^45^, the FePt slab within a fixed 9 monatomic layers (9ML) thickness is considered in all of our calculations. Our main goal is to survey more elements which could conceivably create a higher PMA in FePt slab and thereby to explore the mechanisms enabling these overlayers to enhance or create the interfacial PMA.

## Results and Discussion

### Structure and stability

**Figure 1:**
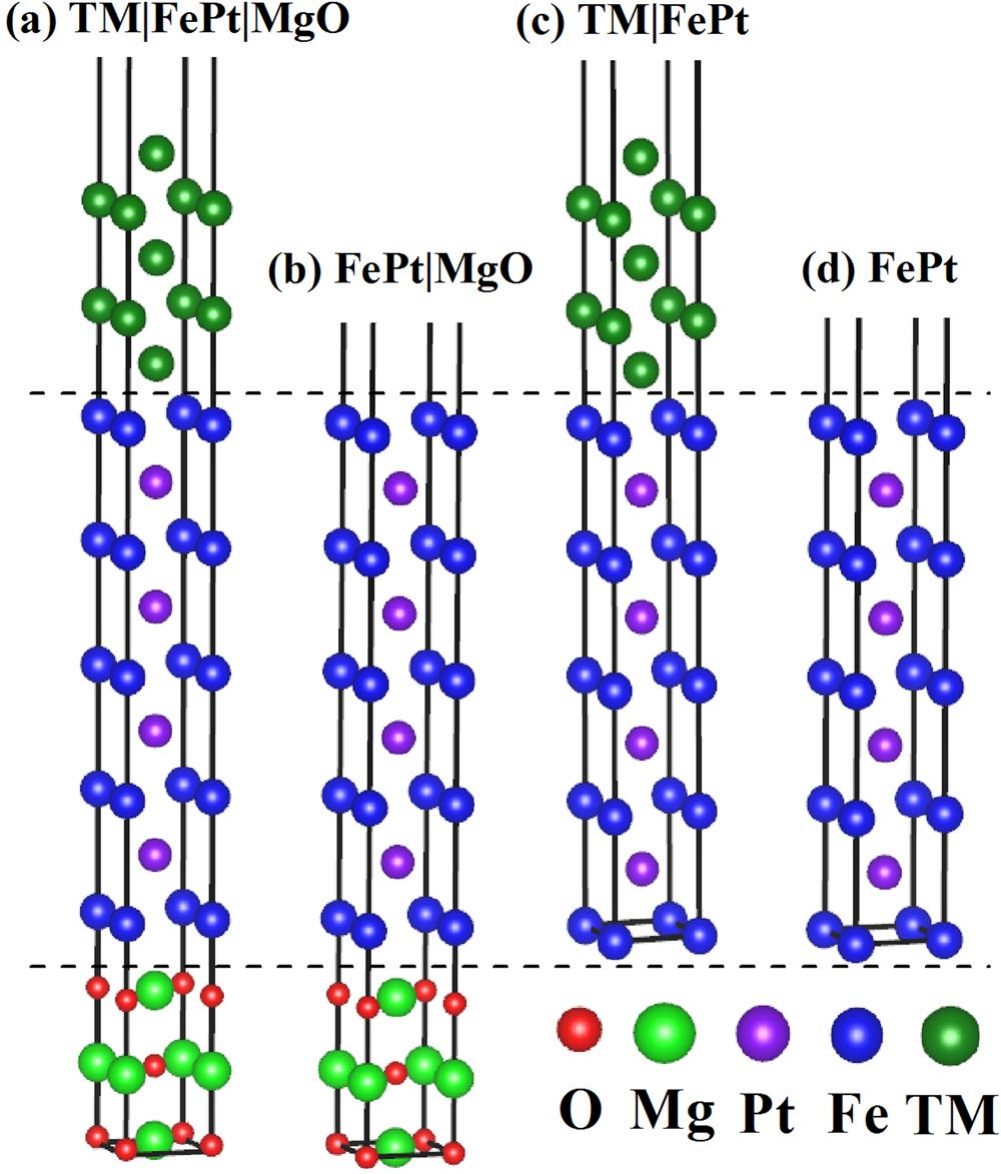
Schematics of crystalline structures for TM|FePt|MgO (**a**), FePt|MgO (**b**), TM|FePt (**c**) and FePt free-standing surface (**d**). Fe-termination layer is adopted and 15 Å vacuum layer is included on top of all the structures.

We have considered the 9ML of *L*1_0_-FePt alloy oriented along the [001] direction, which are sandwiched between 5ML-TM (001) and 3ML-MgO(001) (Fig. 1a). To minimize the artificial interaction between neighboring slabs, a 15 Å vacuum layer is included on top of all the structures. The in-plane lattice constant is constrained at $\sqrt{2}\mathrm{/2}a$ × $\sqrt{2}\mathrm{/2}a$ (experimental values of *L*1_0_-FePt bulk and MgO: *a*_FePt_ = 3.86 Å, *a*_MgO_ = 4.22 Å)^44^, which means that the in-plane lattice of MgO layers is compressed by 8.5% to fit with that of FePt layers. Since the *L*1_0_-FePt in FCT-phase is stacked by alternating Fe and Pt planes, there are Fe- and Pt-terminated contact layers at the FePt|MgO interface, i.e, TM|FePt&hellipsis;PtFe|MgO stacking sequence for Fe-termination (5ML-Fe and 4ML-Pt) while TM|PtFe&hellipsis; FePt|MgO stacking sequence for Pt-termination (4ML-Fe and 5ML-Pt). Regarding the theoretical investigations that the binding interactions at the FePt|MgO interface is much weaker between Fe(Pt) and Mg atoms than between Fe(Pt) and O atoms^43,44^, we only focus on the Fe(Pt) atoms above the O atoms. To well match with the in-plane lattice constants of *L*1_0_-FePt, the latter elements of the Period-Table in the FCC crystalline phase have been considered as the TM capping layers.

Our calculations on the FePt|MgO heterostructure for adsorption energy suggests that the Fe-terminated interface to MgO is more energetically stable than the Pt-terminated one, where the adsorption energy is evaluated by subtracting the energy of clean FePt and MgO alloys from the total energy. By analyzing the interfacial distances *d*(I), it was confirmed that the Fe-termination and the Pt-termination give 2.32 Å and 2.64 Å, respectively, owning to the stronger Fe-O bonding over the Pt-O bonding. These results agree well with previous reports on FePt|MgO heterostructure^43,44^. On the other hand, total energy calculations were conducted on Cu|FePt heterostructure, and the configuration with Cu-Fe contact interface was found to be the lowest energy structure^46^. Consequently, the Fe-terminated interfaces to MgO and/or TM layers, as shown in Fig. 1, are emphasized for the following magnetic discussions unless specified otherwise.

**Figure 2:**
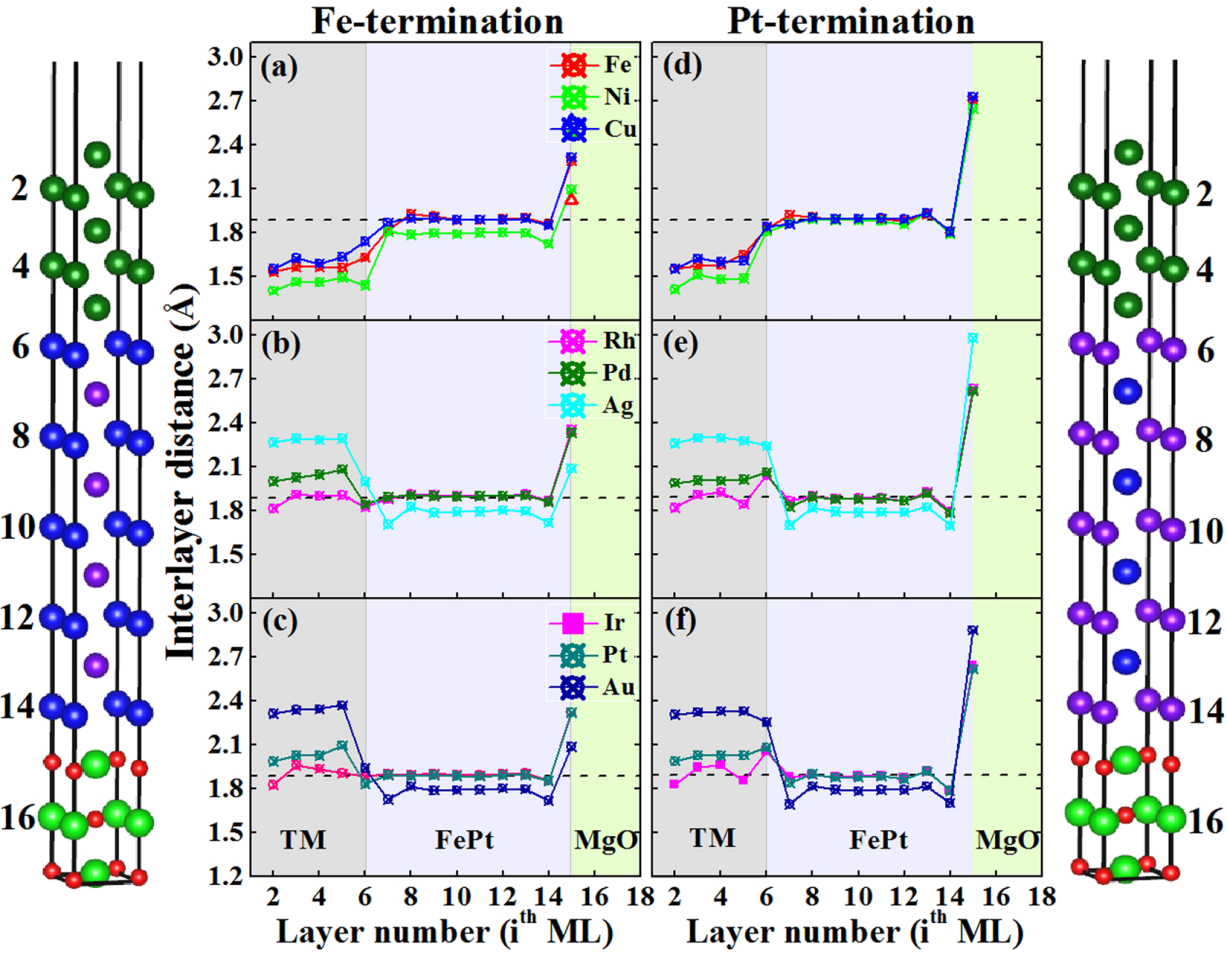
Interlayer distances as the function of the layered orders *i*^th^ML for TM|FePt|MgO heterostructures, where left and right panels are for Fe-terminated and Pt-terminated configurations, respectively. The horizontal dash line indicates the Fe-Pt interlayer distance in *L*1_0_-FePt bulk. Different shaded regions indicate the different stacked-layer slabs.

For our optimized TM|FePt|MgO heterostructures, their interlayer distances as the function of the layered orders *i*^th^ML are given in Fig. 2, where the left- and right-panels are for the Fe-terminated and Pt-terminated interfaces, respectively. It is very intriguing that TM = Ni, Ag, Au capping layers have reduced the interlayer distances between Fe and Pt layers in the FePt slab, compared with the bulk FePt value (dash line). Furthermore, there are significant changes near the TM|FePt and FePt|MgO interfaces (*i*^th^ = 5, 14) for every heterostructure. These results indicate that magnetic moments and PMAs contributed from the FePt slab may be tuned by changing Fe-Pt interlayer distances via the capping of typical TM layers. Since the in-plane lattice constants of bulk Ni, Ag, and Au are 3.52 Å, 4.09 Å, 4.08 Å, respectively, representing the largest mismatches of −9.50%, 5.10%, and 4.13% with FePt among our studied 3*d*, 4*d*, and 5*d* period elements, respectively, the significant relaxations of their in-plane lattice constants should be compensated by the reverse relaxations of their out-of-plane lattice constants so as to stabilize the structural stabilities in TM|FePt|MgO heterostructures. Generally, the larger mismatch and consequently larger in-plane contraction (expansion), the larger out-of-plane expansion (contraction) TM overlayers would exhibit. In this respect, TM interlayer separations near the TM|FePt interface would significantly influence the interfacial interactions between TM-Fe layers, and these interactions would orderly pass on other layers in FePt slab, resulting in the uniform relaxations of Fe-Pt interlayer distances in FePt slab as shown in Fig. 2.

**Figure 3:**
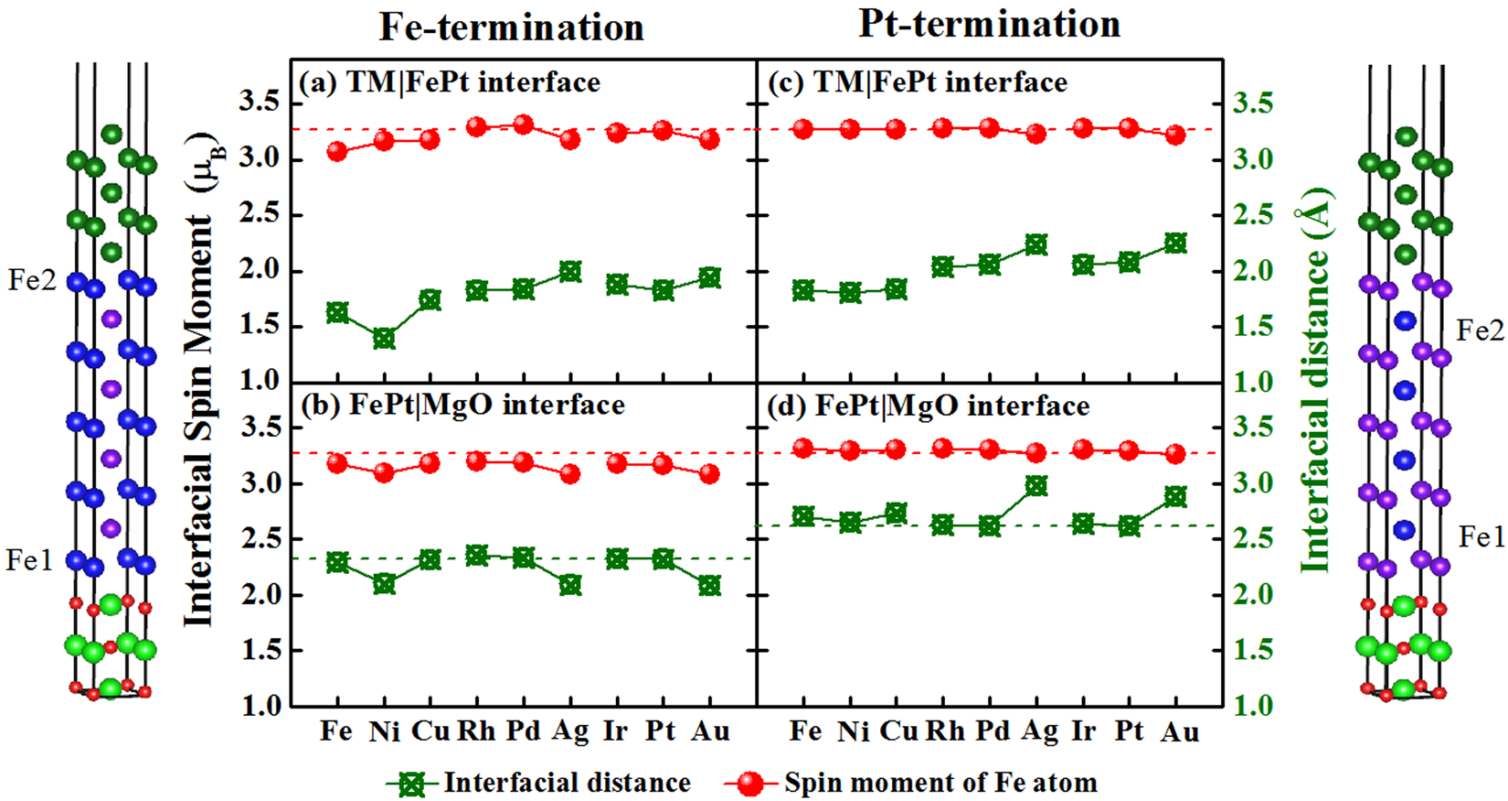
Interfacial Fe spin moment and the interfacial distance at the TM|FePt interface (up-row) and the FePt|MgO interface (down-row) for the Fe-terminated (left) and Pt-terminated (right) configurations in TM|FePt|MgO heterostructures. The red dash line denotes the Fe magnetic moments in bulk FePt, while the green dash line denotes the interfacial distances in FePt|MgO heterostructure without TM capping layers.

To estimate the effect of the TM capping layer on spin magnetic moments of interfacial Fe atoms, the spin moments together with the interfacial distances are plotted as the function of TM element in Fig. 3. Note that Fe atoms in Pt-terminated configurations refer to the sites localized on the sub-interfacial layers. Although TM|FePt|MgO and FePt|MgO heterostructures are constrained at the same in-plane lattice parameter (3.89 Å), from the left-panel, we found that TM|FePt top (TM = Ni, Ag, Au) interfaces can result in slight decreases of interfacial Fe magnetic moments and interfacial distances *d*_Fe−O_ at the FePt|MgO bottom interface, i.e., the stronger Fe-O interfacial interaction, the smaller magnetic moments of interfacial Fe atoms would exhibit. The trend is reverse at the TM|FePt interface, i.e., the longer TM-Fe interfacial distance and thus the weaker interaction, the smaller magnetic moments Fe atoms exhibit. From the right-panel, except for Ag and Au capping layers that largely increase the bottom interfacial Pt-O distances, other TM layers have negligible influence on the properties of FePt|MgO bottom interface. Furthermore, we notice that Fe-O distances are generally smaller than Pt-O ones. This implies that Fe layer will be closer to MgO contact layer, resulting in the complex rearrangement of charge distributions and thus changing the magnetic moments of Fe atoms at the FePt|MgO bottom interface. On the whole, interfacial and interior Fe atoms in different heterostructures maintain the intrinsic spin moments around 3.25 *μ*_*B*_ as Fe atoms exhibit in FePt bulk, which means that spin moments of Fe atoms in FePt slab are negligibly influenced by various TM capping layers.

### Perpendicular magnetocrystalline anisotropy (PMA)

**Figure 4:**
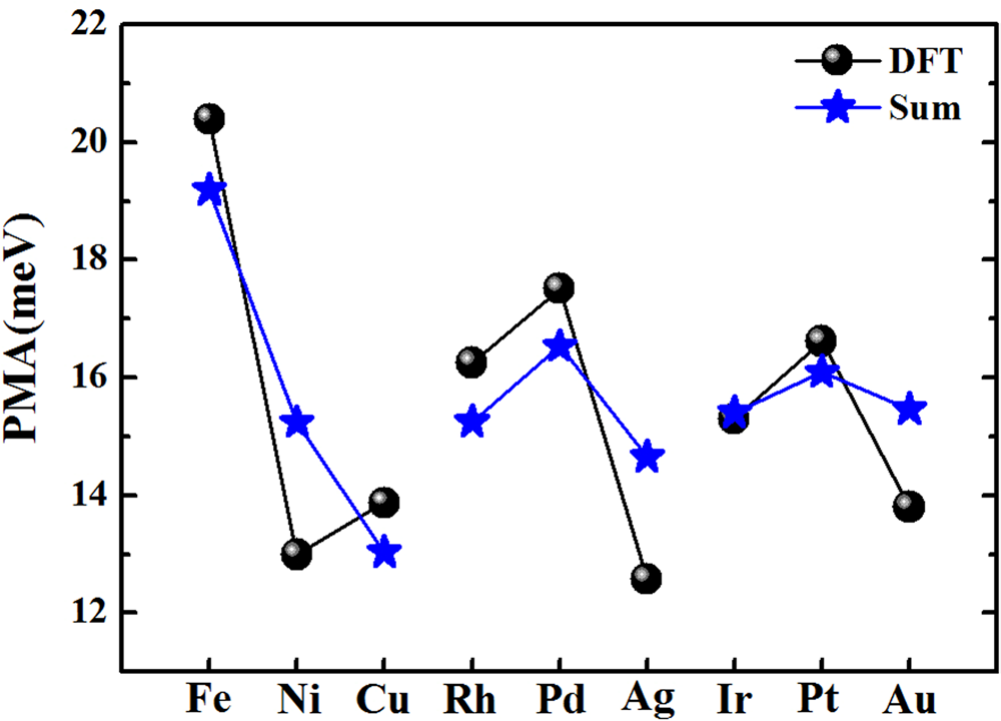
PMAs of TM|FePt|MgO heterostructures calculated from the DFT method and our summing formula.

Our calculated PMA of bulk FePt is 1.85 meV/f.u., which is close to the previous calculation of 1.50 meV/f.u^46^. and experimental value of 1.25 meV/f.u^35^. On the one hand, as 9ML-FePt layers are stacked on MgO substrate within Fe-termination (Fig. 1b), its PMA = 11.90 meV is 3.32 meV larger than the value of FePt free-standing surface within equivalent thickness. This enhancement indicates that the FePt|MgO interface contributes to PMA = 3.32 meV, which can be related to the strong hybridizations between Fe-3*d* and O-2*p* orbitals at FePt|MgO interface^2–7^. On the other hand, as 5ML-TM layers are stacked on 9ML-FePt layers to form TM|FePt heterostructures (Fig. 1c), their PMAs vary from the smallest value of 11.89 meV for Cu capping to the largest value of 17.98 meV for Fe capping (Table 1), suggesting that most TM capping on FePt layers can prompt their PMAs. To assess what extent TM layers contribute to these enhancements, we have calculated the free-standing surfaces within 5ML-TM (TM = Fe, Ni, Cu) at the fixed in-plane lattice constant of TM|FePt|MgO heterostructure, and we found their PMAs are less than 0.1 meV. Therefore, the PMA enhancement in TM|FePt heterostructures comes from the interfacial contributions. Then, we extracted the interfacial PMA by subtracting the PMA of FePt free-standing surfaces from these of TM|FePt heterostructure, and found the values ranging from 3.31 meV to 9.40 meV (Table 1). If we suppose that, in a simple approach, the PMAs of TM|FePt|MgO heterostructures are contributed by two interfaces and interior 7ML-FePt layers, their values can be derived by summing over theses contributions: PMA(TM|FePt|MgO) = PMA(TM|FePt-interface) + PMA(FePt|MgO-interface) + 7 × 0.93, where each FePt layer is approximated to have the bulk value of 0.93 meV/ML. The PMAs obtained from the aforementioned formula and DFT calculations are listed in Table 1 and are compared with each other in Fig. 4. It is clear that they match with each other very well as a function of the elements, exhibiting the same oscillation behavior with maximum values at TM = Fe, Pd, Pt. Note that the Fe|FePt|MgO yields the largest PMA of about 20.4 meV, which is about 2 times larger than 8.58 meV and 8.37 meV of clean free-standing surface and bulk phase within 9ML-FePt, respectively. Based on our extracted interfacial PMA and aforementioned summing approach, the PMA of several previously studied systems have been recalculated to verify the validity of our values. For Pt|Fe|Pt|Fe(001) heterostructure that Fe monolayer was sandwiched by two Pt monolayers and deposited on 10-ML Fe(001) layers, its PMA was calculated to be 7.5 meV by previous DFT method^23^. In terms of our extracted interfacial PMA = 6.37 meV of Fe|FePt interface (Table 1), its PMA(Pt|Fe|Pt|Fe(001)) = 6.37 + 2 × 0.93 = 8.23 meV is only 9.7% larger than their DFT value. For Cu|FePt|MgO heterostructure comprised of 4ML-Cu and 5ML-FePt, our PMA(Cu|FePt|MgO) = PMA(Cu|FePt-interface) + PMA(FePt|MgO-interface) + 3 × 0.93 = 3.31  +  3.32 + 2.79 = 9.42 meV is 11.7% less than the previous calculation of 11.88 × 10^7^ ergs/cm^3^ (≈10.67 meV)^45^. Therefore, we can deduce that our extracted interfacial PMA is reasonable and reliable, and appropriate TM capping would enhance the PMA of FePt-based heterostructures through the TM|FePt interface.

**Table 1:** The DFT calculated PMAs (meV) for different heterostructures within the Fe terminations.

System(Fe-termination)	3*d*			4*d*			5*d*		
	Fe	Ni	Cu	Rh	Pd	Ag	Ir	Pt	Au
TM|FePt(DFT)	17.98	14.10	11.89	14.11	15.39	13.51	14.27	14.95	14.32
TM|FePt|MgO(DFT)	20.39	12.99	13.87	16.25	17.51	12.57	15.30	16.62	13.80
TM|FePt|MgO(Sum)	19.19	15.24	13.03	15.25	16.53	14.65	15.41	16.09	15.46
FePt|MgO Interface(Extracted)	3.32	—	—	—	—	—	—	—	—
TM|FePt Interface(Extracted)	9.40	5.52	3.31	5.53	6.81	4.93	5.69	6.37	5.74

To validate our extracted interfacial PMAs at the FePt|MgO and TM|FePt interfaces, their summed values in addition to that of interior 7ML-FePt layers are compared with the DFT values for each TM|FePt|MgO heterostructure.

**Figure 5:**
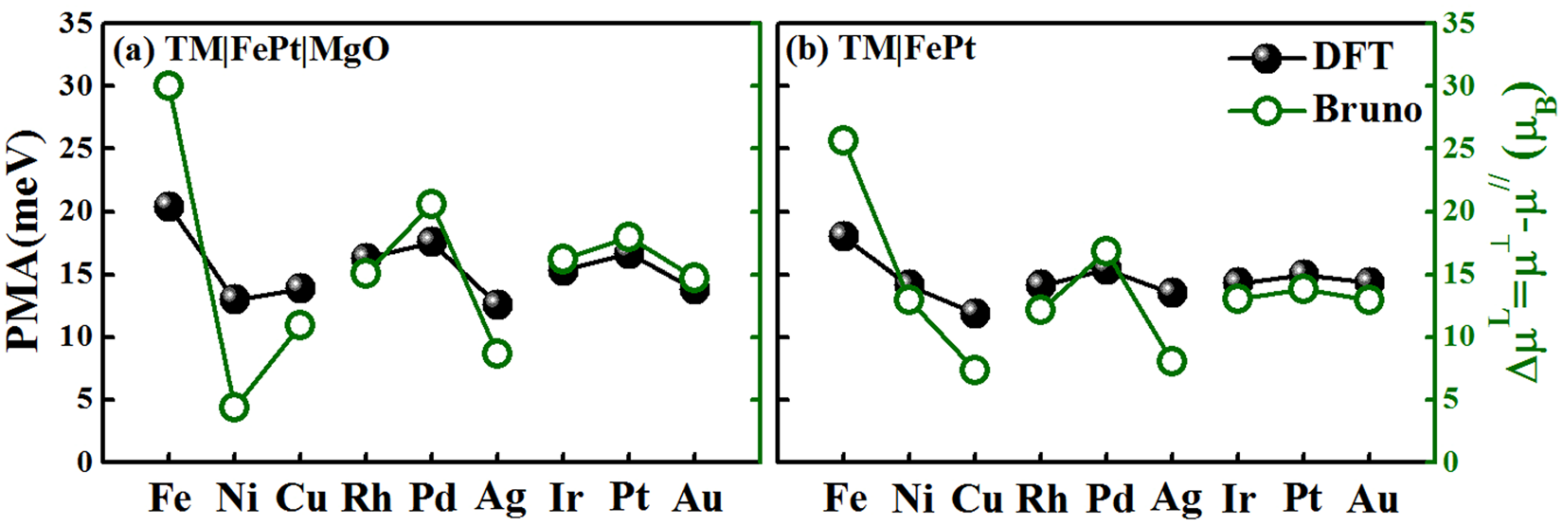
PMAs of TM|FePt|MgO and TM|FePt heterostructures calculated from the DFT method and Bruno's formula PMA≃−*ξ*Δ*μ*^L^/4*μ*_*B*_.

The model that relates the orbital moment has been developed and employed successfully to establish a phenomenological understanding of the magnetic anisotropy. On the basis of the second-order perturbation theory, Bruno has proposed a formula, i.e., PMA≃−*ξ*Δ*μ*^L^/4*μ*_*B*_^48,49^, where *ξ* is SOC constant and Δ*μ*^L^ = ${\mu }_{001}^{{\rm{L}}}-{\mu }_{100}^{{\rm{L}}}$ is orbital moment anisotropy. In Fig. 5, the PMAs calculated within Bruno's formula are compared to the values calculated within DFT calculations for TM|FePt|MgO (left-panel) and TM|FePt (right-panel) heterostructures, respectively, where *ξ* = −300 meV is employed by fitting the variational curves with each other. The fitted *ξ* is between our calculated values of −10 meV and −824 meV for Fe and Pt atoms. Two analogous oscillating trends demonstrate that the Bruno's relationship can be applicable for FePt-based heterostructures. Note that the Bruno's formula is just a model calculation that has certain approximations built in, and it aims to qualitatively illustrate the relationship between the PMA and the orbital moment. Hence, one has to be aware that in some cases the accuracy of the results is limited. For example, *ξ* is not a spin-orbital coupling constant of elemental atoms but an adjustable parameter when applicable for alloyed magnetic systems.

### Effect of in-plane strain on PMA

**Figure 6:**
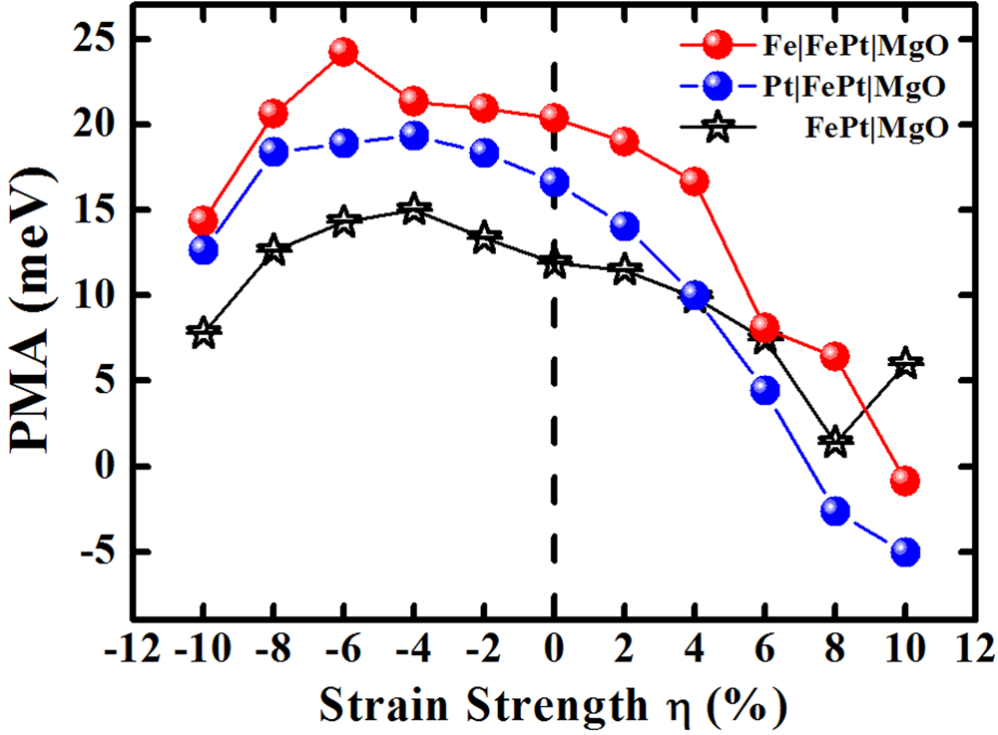
PMAs of TM|FePt|MgO (TM = Fe, Pt) heterostructures as a function of the strain strength *η*. The variational trend of FePt|MgO heterostructure is shown for comparison.

To explore the in-plane strain effect on the PMA, we have varied the in-plane lattice constants *a* of TM|FePt|MgO and FePt|MgO heterostructures. The strain strength *η* is defined as *η* = (*a*−*a*_0_)/*a*_0_ × 100%, with *a*_0_ = 3.89 Å and *a* = 3.50~4.28 Å being the in-plane lattice constant without and with the strain, respectively. The minimum (maximum) value is close to the optimized lattice constant of TM (MgO) crystalline bulk under our GGA calculations. According to the changing *a*, the interlayer spacings will vary too, so the out-of-plane lattice constant *c* has been optimized for each in-plane lattice value. Taking Fe|FePt|MgO and Pt|FePt|MgO heterostructures as the representative systems, we demonstrated in Fig. 6 that the PMA can be tuned in a large range by the in-plane strain. Although the easy axis is always retained in perpendicular magnetization, there are increasing trends followed by decreasing trends with the increasing lattice constants. The similar behavior has been observed in single crystal (Co, Fe)Pt, and FePd bulk^50,51^, as well as FePd|MgO heterostructures^52^.

Under the negative strain, Fe|FePt|MgO and Pt|FePt|MgO heterostructures present a similar increasing trend and their PMAs uniformly achieve to the maximum values at *η* = −(4~6)%. At every strain strength, Fe and Pt capping layers have enhanced the PMAs of FePt|MgO by near constant values of 8 meV and 6 meV, respectively. For FePt|MgO heterostructure, the variational trend can be interpreted as following reasons: (i) With the contraction of the in-plane lattice constants (elongation of the out-of-plane constants), the decreased interactions between the surfacial Fe atoms and the sub-surfacial Pt atoms enable more Fe-3*d* electrons localized on their minority-spin orbitals, because their Fe-3*d* majority orbitals are fully occupied. These Fe-3*d* minority-spin electrons would lead to more unbalanced distributions of the projected occupation numbers of Fe-3*d* electrons onto their sub-orbitals (*m* = 0, ±1, ±2), increasing the surfacial orbital moments and enhancing the surfacial PMA. (ii) The weakened Fe(3*d*)-Pt(5*d*) hybridization would reduce the intrinsic PMAs of interior 7ML in FePt slab. (iii) Although the elongated FePt|MgO interfacial distance and consequently the weakened O(2*p*)-Fe(3*d*) hybridization also decreases the interfacial PMA, the less transferred Fe-3*d* minority electrons would increase the interfacial PMA to some extent. Once the compression strain is beyond a certain value *η*<−6%, the negative contributions would overcompensate the positive contributions, resulting in the gradually decreasing trends. Under the positive strain, theses deductions are also suitable to the interpretations of decreasing trend. Nonetheless, the PMA is robust for FePt|MgO heterostructure under any strains.

For TM|FePt|MgO heterostructure, there is an additional TM|FePt interface yet an eliminated FePt surface. With the increasing strain *η* > 0, this interfacial PMA undergoes the gradual reduction as FePt surface does, which leads to the similar variational trends of FePt|MgO and TM|FePt|MgO. At a given strain, this interfacial reduction makes TM|FePt|MgO heterostructure decrease their total PMAs more largely than the surface of FePt|MgO heterostructure does. Consequently, FePt|MgO and Pt|FePt|MgO (Fe|FePt|MgO) heterostructures present an intersection between two curves at *η* = 4%(6%). The magnetization changes from perpendicular to in-plane when the stretching strain is large enough for TM|FePt|MgO heterostructures (PMA changes from the positive to negative value). In other words, the strain in such systems can introduce the changes in the 3*d*-orbital occupations and also lead to the movements of energy levels of 3*d* orbitals, resulting in the variations of the PMAs.

### The origin of magnetic moment and PMA

The perpendicular magnetic anisotropy (PMA) is related to the spin-orbit coupling (SOC) interaction induced splitting and shifting of the degenerate electronic states which depend on the magnetization direction. Generally speaking, the PMA can be separated into the volume, surface, and interface contributions, all of which are originated from the interlayer hybridization, i.e., strong SOC interaction between the magnetic and nonmagnetic metals. The Fe(Co)-3*d* and O-2*p* hybridization at the Fe(Co)|MgO interface^16,20^ as well as the Fe(Co)-3*d* and Pt-5*d* hybridization at the Fe(Co)|Pt interface^16–21^ and in *L*1_0_-FePt bulk^37^ belong in this category. Experimentally, on the one hand, heavy-metals including TM = Pt, Pd, W, Hf, Ta within large SOC strength were extensively explored to deposited on Fe(Co) layers to create the Fe(Co)-TM bonding at the Fe(Co)|TM interface^18,22,25–30,47^, where large PMA and orbital moments of Fe(Co) atoms are magnetically induced by 5*d*-TM atoms through the strong 3*d*-5*d* hybridization^53^. On the other hand, MO_*x*_(M = Mg, Al, etc) layers were intensively utilized as the underlayers to develop the Fe(Co)-O bonding at the Fe(Co)|MO_*x*_ interface^54,55^, where large PMA and orbital moments of Fe(Co) atoms on MO_*x*_ arise from the interplay between SOC and low-symmetry ligand field of the O adsorption site^56^. In short, the combination of strong SOC strength, large orbital moment and appropriate ligand field plays an essential role for magnetic systems in realizing large PMA. Up to now, there are several favoured ways to achieve this objective by changing the band filling of *d* orbitals of ferromagnetic layer, including the heavy-metal capping, appropriate underlayer supporting, lattice-mismatch induced strain and electric field assistant.

**Figure 7:**
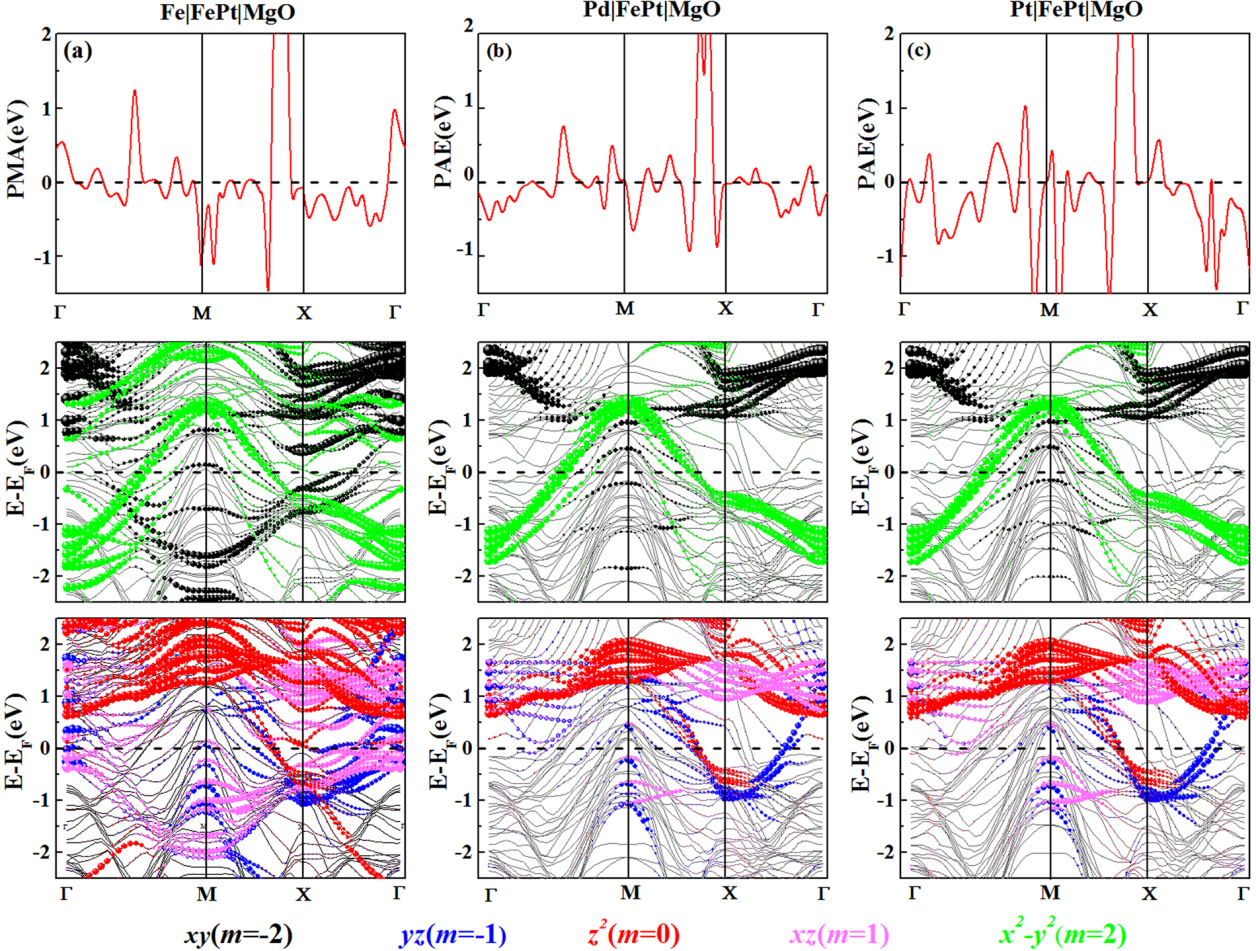
PMA(*k*) contributions along high-symmetry directions in the two-dimensional Brillouin zone for Fe|FePt|MgO (**a**), Pd|FePt|MgO (**b**), and Pt|FePt|MgO (**c**) are shown on the top row. The corresponding energy- and *k*-resolved distribution of the orbital character of the minority-spin band of 9ML-FePt slab for Fe *d*_*xy*_ and ${d}_{{x}^{2}-{y}^{2}}$ (middle row), *d*_*yz*_, ${d}_{{z}^{2}}$ and *d*_*xz*_ (bottom row). The *d*_*xy*_, *d*_*yz*_, ${d}_{{z}^{2}}$, *d*_*xz*_ and ${d}_{{x}^{2}-{y}^{2}}$ orbital states are coloured by the black, blue, red, magenta, and green balls, respectively. The sizes of ball stand for the amplitude of the corresponding orbital character.

Here, to elucidate the electronic origination of the PMA, we have taken TM = Fe, Pd, Pt capping layers as the typical systems and decomposed their PMA(*k*) distributions in the two-dimensional Brillouin zone (BZ) on the top row Fig. 7(a–c). The corresponding band structures projected onto five *d* orbitals for the minority-spin state of Fe atoms are shown in the middle and bottom rows, respectively. The PMA arising from the SOC interaction is formulated in the framework of second-order perturbation as PMA ≃ ${\xi }^{2}{\sum }_{o,u}$ $\frac{| < {{\rm{o}}}^{\sigma }|{l}_{z}|{{\rm{u}}}^{\sigma ^{\prime} } > {|}^{2}-| < {{\rm{o}}}^{\sigma }|{l}_{x}|{{\rm{u}}}^{\sigma ^{\prime} } > {|}^{2}}{{\varepsilon }_{u{\rm{\sigma }}^{\prime} }-{\varepsilon }_{o{\rm{\sigma }}}}$^57^, where o^σ^(u^σ′^) and *ε*_o*σ*_(*ε*_u*σ*′_) represent the eigenstates and eigenvalues of the occupied (unoccupied) states of magnetic atoms in the spin state *σ*(*σ*′) and *ξ* is the SOC constant. The total PMA is the summed values over all atoms in the unit cell. Since the majority part of the Fe-3*d* band is fully occupied and well below the Fermi energy in various FePt systems, the SOC interactions between the majority spin states as well as between the opposite spin states are negligible. The main attributions to the PMA come from the interaction between states in the minority Fe-3*d* band near the Fermi level, i.e., the positive contributions to the PMA are characterized by *l*_*z*_ operator via <*xz*|*l*_*z*_|*yz*> = 1, <*xy*|*l*_*z*_|*x*^2^−*y*^2^> = 2; the negative contributions are characterized by *l*_*x*_ operator via <*z*^2^|*l*_*x*_|*xz*,*yz*> = $\sqrt{3}$, <*xy*|*l*_*x*_|*xz*, *yz*> = 1, and <*x*^2^ − *y*^2^|*l*_*x*_|*xz*, *yz*> = 1. Here, we expressed the 3*d*-orbitals as ${d}_{{z}^{2}}$, *d*_*xz*/*yz*_, ${d}_{{x}^{2}-{y}^{2}/xy}$ with their magnetic quantum numbers *m* = 0, *m* = ±1, *m* = ±2, respectively.

In Fig. 7(a) for Fe|FePt|MgO, the PMA(*k*) exhibits two dominant positive peaks around 1/2(Γ-M) and X points and two negative peaks around M and 2/3(M-X) points. The positive values are related to the coupled contributions between the occupied *m* = 2 states and the unoccupied *m* = −2 states, because their reduced energy differences *ε*_u↓_−*ε*_o↓_ around *k* points could enhance their positive PMA via <*m* = 2|*l*_*z*_|*m* = −2> matrix. The negative values are dominated by the contribution from <*m* = 0|*l*_*x*_|*m* = ±1> and <*m* = 2|*l*_*x*_|*m* = ±1> matrixes. In Fig. 7(b) for Pd|FePt|MgO, similar PMA(*k*) trend together with analogous orbital contributions can be derived, except for the evidence that the PMA(*k*) is approximated to be zero around the M point. The *m* = −2 state shifts up and localizes near the Fermi level with respect to the states of Fe|FePt|MgO, which results in the modest enhancement of positive contribution via <*m* = 2|*l*_*z*_|*m* = −2> matrix and thus offsets the negative contribution from <*m* = 2|*l*_*x*_|*m* = ±1> matrix. In Fig. 7(c) for Pt|FePt|MgO, there presents one prominent positive peak around the X point through the positive contribution from <*m* = 2|*l*_*z*_|*m* = −2> matrix. Nevertheless, negative contributions to PMA around 1/2(Γ−M) become much stronger due to the emergence of <*m* = 2|*l*_*x*_|*m* = ±1> matrix.

**Figure 8:**
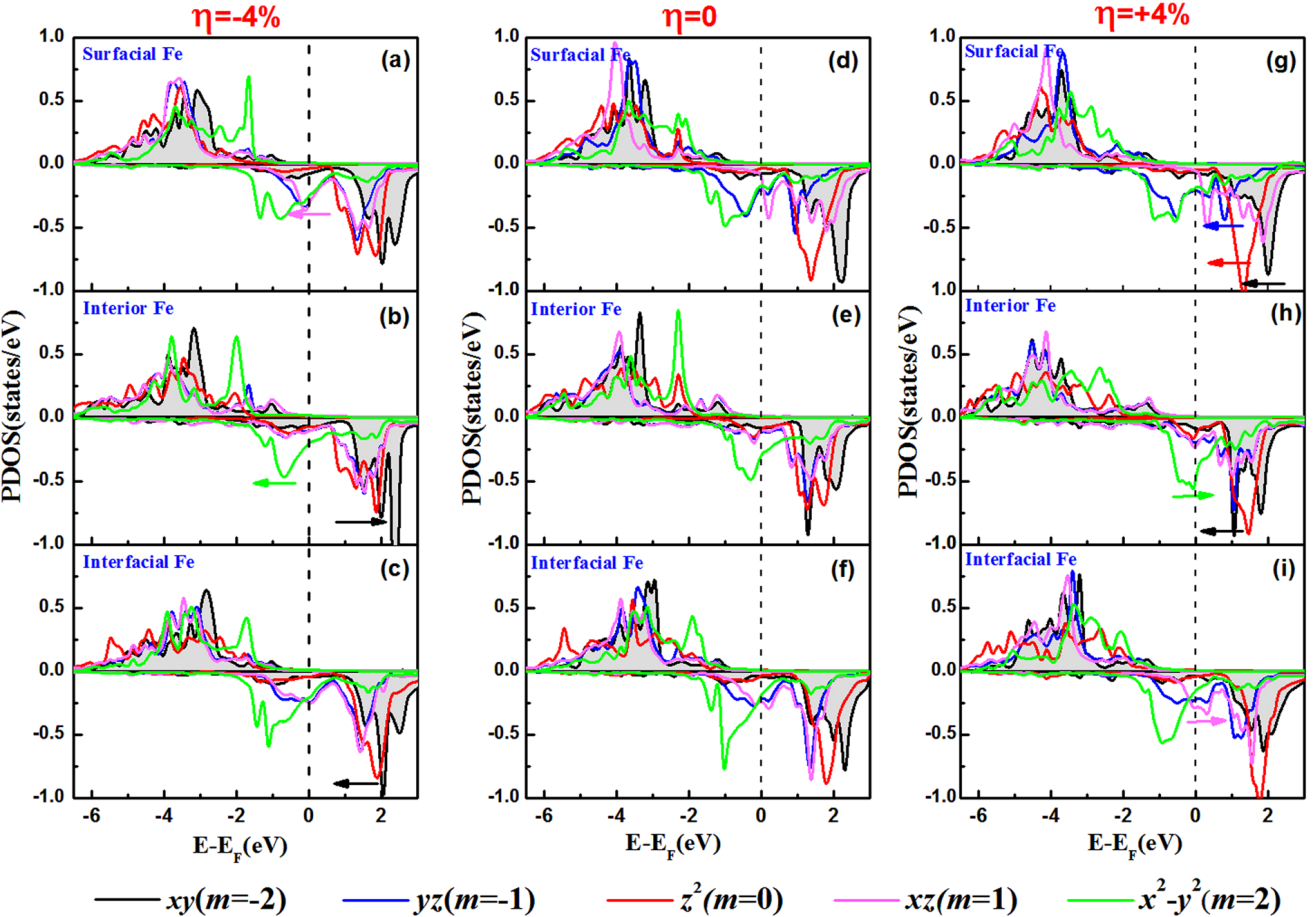
The 3*d*-orbital resolved projected density of states (PDOS) for the surfacial-Fe atoms (**a**), interior-Fe atoms (**b**), interfacial-Fe atoms (**c**) in FePt|MgO heterostructure under *η* = −4% (left-panel); (**d**–**f**) for the counterpart Fe atoms under *η* = 0 (middle-panel); (**g**–**i**) for the counterpart Fe atoms under *η* = +4% (right-panel). The *d*_*xy*_, *d*_*yz*_, ${d}_{{z}^{2}}$, *d*_*xz*_ and ${d}_{{x}^{2}-{y}^{2}}$ orbital states are shown in black, blue, red, magenta and green lines, respectively. The Fermi level is set to zero energy.

To clarify the PMA modification with the strain strength, in Fig. 8 we presented the 3*d*-orbital resolved projected density of states (PDOS) for the surfacial-Fe atoms (a), interior-Fe atoms (b), interfacial-Fe atoms (c) under *η* = −4% (left-panel); (d–f) for the counterpart Fe atoms under *η* = 0 (middle-panel); (g–i) for the counterpart Fe atoms under *η* = +4% (right-panel) in FePt|MgO heterostructure. Hereafter, the movements of the mentioned peaks are relative to the peak positions under *η* = 0. From the left-panel for the compression *η* = −4%, *m* = 1 states of the surfacial-Fe atoms move below the Fermi level (a), giving an additional large positive PMA via <*m* = 1|*l*_*z*_|*m* = −1> matrix; *m* = 2 and *m* = −2 states of the interior-Fe atoms move toward the lower and higher energy levels, respectively (b), reducing the positive PMA via <*m* = 2|*l*_*z*_|*m* = −2> matrix due to their increased energy difference *ε*_u↓_-*ε*_o↓_; *m* = −2 states of the interfacial-Fe atoms move toward the lower energy levels (c), slightly increasing the positive contribution and consequently increasing PMA via <*m* = 2|*l*_*z*_|*m* = −2> matrix. In other words, the movements of orbital positions suggest the variations of the orbital occupation number, which results in the changes of the magnitude of SOC matrixes and naturally brings the changes of PMA. On the whole, opposite contributions from surfacial-Fe, interior-Fe, and interfacial-Fe atoms in FePt|MgO heterostructure lead to the net increased PMA under *η* = −4% with respect to the value under *η* = 0.

From the right-panel for the expansion *η* = +4%, *m* = 0 and −1 states of the surfacial-Fe atoms shift toward the lower energy (g), which reduces the total PMAs because of the increased negative contributions via <*m* = 2|*l*_*x*_|*m* = −1> and <*m* = −1|*l*_*x*_|*m* = 0> matrix, respectively; *m* = 2 and *m* = −2 states of the interior-Fe atoms move toward the higher and lower energy levels, respectively (h), increasing the positive PMA via <*m* = 2|*l*_*z*_|*m* = −2> matrix; *m* = 1 states around the Fermi level of the interfacial-Fe atoms shift upward (i), reducing the positive PMA via <*m* = −1|*l*_*z*_|*m* = 1> matrix. The reduced positive contributions from surfacial-Fe and interfacial-Fe atoms overcompensate the increased positive contributions from interior-Fe in FePt|MgO heterostructure, leading to the net decreased PMA under *η* = +4% with respect to the value under *η* = 0. From Fig. 8, it is general that the SOC coupling between the occupied *m* = −2 and unoccupied *m* = +2 orbital always leads to large PMA of TM|FePt|MgO heterostructures. This coupling, <*m* = ±2|*l*_*z*_|*m* = ±2>, has the largest contribution to the PMA by a factor of 2 among all effective matrix elements^57^.

**Figure 9:**
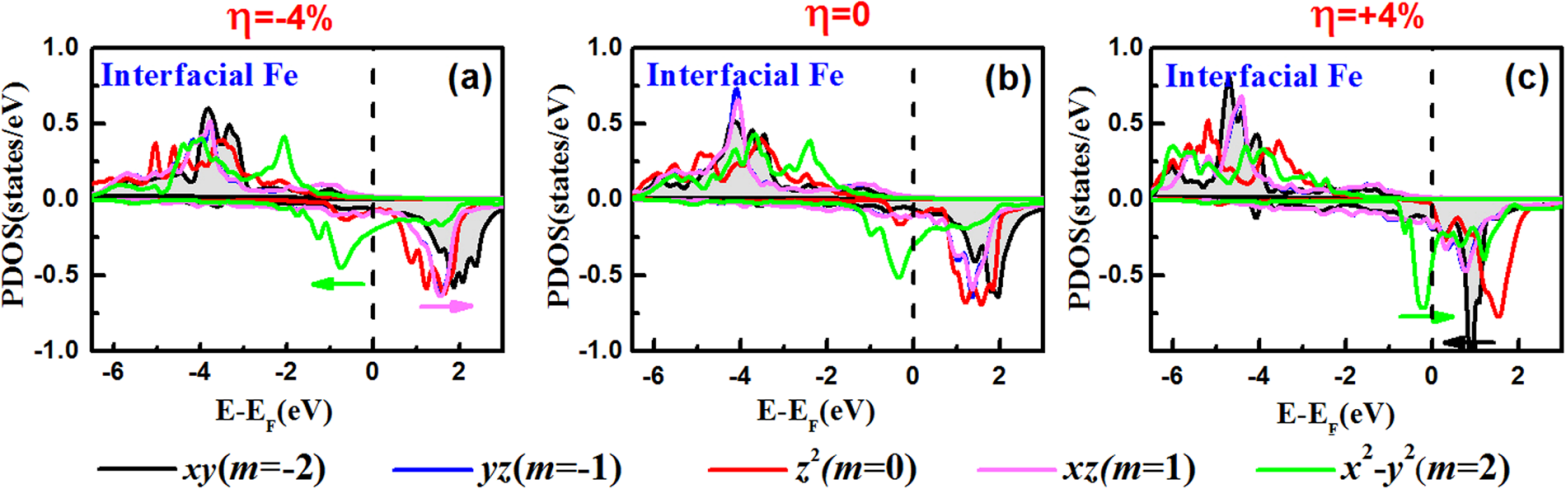
Same as in Fig.8 but for interfacial Fe atoms at the Pt|FePt interface in Pt|FePt|MgO.

Finally, to reveal the effect of TM capping layers on the PMA modification, Fig. 9 shows the 3*d*-orbital resolved PDOS of the interfacial Fe atoms at the Pt|FePt interface in Pt|FePt|MgO. At the strain-free condition *η* = 0, it is clear that the positive contribution to out-of-plane PMA is determined by the SOC interaction between occupied *m* = 2 and unoccupied *m* = −2 states. Consequently, the TM|FePt interface shows large PMA. Under the in-plane compression *η* = −4%, the energy difference of the peaks between the occupied *m* = 2 states and unoccupied *m* = 1 states increases slightly, which means that the negative contribution via <*m* = 2|*l*_*x*_|*m* = 1> matrix would be reduced and the total PMA would be increased. Under the expansion *η* = +4%, the peaks of the occupied *m* = 2 and unoccupied *m* = −2 states move close to each other, which increases the primarily positive PMA contributions through <*m* = 2|*l*_*z*_|*m* = −2>. Nevertheless, it seems that this conclusion is in contrary to the results shown in Fig. 6, where the positive strain leads to a decrease in PMA of TM|FePt|MgO. In fact, the strain would influence the interfacial PMA not only for TM|FePt top-interface but also for FePt|MgO bottom-interface. Under the expansions of in-plane lattice constant, the contractions of the interlayer spacings lead to strong Pt-Fe interfacial interactions and make this interfacial Pt layer be a part of interior FePt slabs (interfacial distance 1.74 Å is less than interior distance 1.83 Å between Fe-Pt layers), giving the positive contribution to PMA at the top-interface; on the contrary, it gives the negative contribution to PMA at the bottom-interface as analyzed in Fig. 8. Ultimately, the negative contributions overcompensate for the positive contributions, resulting in the net decreased PMA shown in Fig. 6.

## Summary

In summary, we have carried out the systemical DFT calculations on TM|FePt|MgO heterostructures (3*d*-TM: Fe, Ni, Cu; 4*d*-TM: Rh, Pd, Ag; 5*d*-TM: Ir, Pt, Au) for their geometric structures, magnetic moments, and magnetic anisotropy. The Fe-termination is found to be more stable than Pt-termination in constructing the TM|FePt|MgO heterostructures. The TM adlayers having larger in-plane mismatch with the underneath FePt layers can modify the Fe-Pt interlayer spacings and even modulate the Fe-O distance at the FePt|MgO interface. With respect to the corresponding values of FePt bulk and FePt|MgO heterostructure, the TM capping layers paly a negligible effect to alter the magnetic moments of FePt slab, however, they always lead to the significant enhancements of PMA due to the strong and robust contributions of the interfacial PMA at the TM|FePt interfaces. We found that the PMA changes dramatically by varying the TM capping layers, and they can be dramatically modulated with the in-plane strain. Using the second-order perturbation method with electronic structural analyses, we revealed that the PMAs of TM|FePt|MgO heterostructures, including the contributions from TM|FePt interface, FePt interior layers, and FePt|MgO interface, are dominantly contributed by the Fe-3*d* orbitals through the SOC interactions between the occupied ${d}_{{x}^{2}-{y}^{2}}$ and the unoccupied *d*_*xy*_ states. This work can significantly benefit the promotion of the PMAs in the FePt-based heterostructures through the choice of a proper contact overlayers, suggesting the possibilities of the realisations of TM|FePt|MgO heterostructures as the novel spintronic devices and the next generation of ultrahigh-density storage devices.

## Method of Calculations

All of the calculations have been carried out with the density function theory (DFT) method as implemented in VASP code^58–60^. The projector augmented wave (PAW)^61,62^, together with the plane-wave cutoff energies of 500 eV, was adopted to treat the ion-electron interaction. For the exchange-correlation functional, we employed the Perdew, Burke, and Ernzerhof (PBE) function of the generalized gradient approximation (GGA)^63^. To improve the convergence of the solution of the self-consistent Kohn-Sham equations, the discrete energy levels were broadened by using the first Methfessel-Paxton (MP) method with a smearing parameter of *σ* = 0.05 eV. The three step method was utilized to calculate the PMA: (1) The structural optimizations were firstly performed under the scalar-relativistic pseudopotential, where the Monkhorst-Pack *k*-point meshes of 8 × 8 × 1, residual force less than 0.03 eV/Å, and total energy converging criterion of 10^−5^ eV were adopted. During the minimization for each materials, all these structures were fully relaxed. (2) The static self-consistent field (SCF) calculations based on the equivalent structures were then executed without the SOC treatment, where more dense *k*-point meshes of 10 × 10 × 1 and more rigid energy criterion of 10^−6^ eV were adopted to get an accurate charge density distribution. (3) Two fully relativistic non-SCF calculations including the SOC interaction are respectively determined as the magnetization orientations are orderly along the in-plane and out-of-plane directions, where the obtained charge density was read and it remained constant during the calculations. The energy difference ${\rm{\Delta }}E={E}_{100}^{\Vert }-{E}_{001}^{\perp }$ between these two magnetization directions gives PMA, i.e., positive values stand for the PMA and the easy-axis is normal to the basel plane of the FePt (001) surface.
